# Frequent overexpression of HMGA1 and 2 in gastroenteropancreatic neuroendocrine tumours and its relationship to *let-7* downregulation

**DOI:** 10.1038/sj.bjc.6604883

**Published:** 2009-01-20

**Authors:** M M Rahman, Z R Qian, E L Wang, R Sultana, E Kudo, M Nakasono, T Hayashi, S Kakiuchi, T Sano

**Affiliations:** 1 Department of Pathology, Institute of Health Biosciences, University of Tokushima Graduate School, Tokushima, Japan; 2 Department of Pathology, Faculty of Medicine, Kagawa University, Takamatu, Japan; 3 Department of Internal Medicine and Molecular Therapeutics, Institute of Health Biosciences University of Tokushima Graduate School, Tokushima, Japan

**Keywords:** GEP NETs, HMGA1, HMGA2, *let-7*

## Abstract

The molecular pathogenesis of gastroenteropancreatic (GEP) neuroendocrine tumours (NETs) remains to be elucidated. High-mobility group A (HMGA) proteins play important roles in the regulation of transcription, differentiation, and neoplastic transformation. In this study, the expression of HMGA1 and HMGA2 was studied in 55 GEP NETs. Overexpression of HMGA1 and 2 was frequently detected in GEP NETs compared with normal tissues. Nuclear immunostaining of HMGA1 and 2 was observed in GEP NETs (38 of 55, 69%; 40 of 55, 73%, respectively). High-mobility group A2 expression increased from well-differentiated NET (WNET) to well-differentiated neuroendocrine carcinoma (WNEC) and poorly differentiated NEC (PNEC) (*P*<0.005) and showed the highest level in stage IV tumours (*P*<0.01). In WNECs, the expression of HMGA1 and 2 was significantly higher in metastatic tumours than those without metastasis (*P*<0.05). Gastroenteropancreatic NETs in foregut showed the highest level of HMGA1 and 2 expressions. MIB-1 labelling index (MIB-1 LI) correlated with HMGA1 and 2 overexpression (*R*=0.28, *P*<0.05; *R*=0.434, *P*<0.001; respectively) and progressively increased from WNETs to WNECs and PNECs (*P*<0.001). *Let-7* expression was addressed in 6 normal organs, 30 tumour samples, and 24 tumour margin non-tumour tissues. Compared with normal tissues, *let-7* downregulation was frequent in NETs (19 of 30, 63%). Higher expression of HMGA1 and 2 was frequently observed in tumours with *let-7* significant reduction (53, 42%, respectively). The reverse correlation could be detected between HMGA1 and *let-7* (*P*<0.05). Our findings suggested that HMGA1 and 2 overexpression and *let-7* downregulation might relate to pathogenesis of GEP NETs.

Although the gastrointestinal (GI) tract and pancreatic organ have the largest population of endocrine cells, gastroenteropancreatic (GEP) neuroendocrine tumours (NETs) are rare with an age-adjusted annual incidence of 2.5–4.5 per 100 000 (Maggard *et al*, 2004). However, their incidence is increasing by approximately 6% per year (Gustafsson *et al*, 2008). Gastroenteropancreatic NETs comprise a heterogeneous group of neoplasms arising from the diffuse neuroendocrine system and are divided by their embryological site of origin into foregut, midgut, and hindgut tumours (Maggard *et al*, 2004). These tumours strongly differ from each other on the basis of different pathogenetic, clinical, functional, histological, and prognostic patterns (Klöppel *et al*, 2004). The molecular mechanisms of GEP NETs tumourigenesis are poorly understood (Zikusoka *et al*, 2005).

The high-mobility group A (HMGA) proteins are non-histone chromosomal proteins that bind through their AT-binding motifs to the minor groove of AT-rich DNA strands. They have no intrinsic transcriptional activity but can modulate transcription by altering chromatin architecture (Reeves and Nissen, 1990; Thanos and Maniatis, 1992; Thanos *et al*, 1993). The two types of HMGA protein, HMGA1 and HMGA2, have similar functions and are encoded by two different genes at chromosomal loci 6p21.3 and 12q15, respectively (Johnson *et al*, 1989). High-mobility group A proteins are widely expressed during embryogenesis, whereas their expression is low or absent in normal adult tissues (Zhou *et al*, 1995; Chiappetta *et al*, 1996). High-mobility group A proteins are involved in many diverse biological processes such as regulation of transcription, embryogenesis, differentiation, neoplastic transformation, and integration, and expression of viral genomes (Sgarra *et al*, 2004). Both HMGA1 and 2 proteins are overexpressed in several experimental and human malignancies. Overexpression of HMGA1 has been noted in different cancer types and it has been recognised as a good target for therapeutic intervention in pancreatic cancer. High-mobility group A2 overexpression is a hallmark of various benign and malignant tumours and is also associated with a highly malignant phenotype and is a poor prognostic index (Fusco and Fedele, 2007). However, there is no study to detect HMGA proteins expression and to evaluate their role in GEP NETs.

The microRNAs (miRNA), a family of mature noncoding small RNA 21–25 length nucleotides, have been shown to play a role in a variety of biological processes. They regulate gene expression at the posttranscription level by direct cleavage of a target mRNA using interference machinery (mRNA cleavage) or by inhibition of protein synthesis (Caldas and Brenton, 2005; Esquela-Kerscher and Slack, 2006). Recent evidence has shown that miRNA misexpression correlates with various human cancers and indicated that some miRNAs can function as oncogenes or tumour suppressors (Esquela-Kerscher and Slack, 2006). Several studies reported that HMGA2 is negatively regulated by the *let-7* miRNA family in a mouse model system (Mayr *et al*, 2007), in head and neck cancers (Hebert *et al*, 2007), in uterine leiomyomas (Wang *et al*, 2007), in lung cancer cell lines (Lee and Dutta, 2007), and in ovarian cancers (Shell *et al*, 2007). By computer searches (TargetScan, TargetScanS, PicTar, and miRNAviewer), HMGA1 can also be potentially regulated by *let-7*. To our knowledge, there are few reports about miRNA expression in GEP NETs and no investigation about the relations between HMGA1 and 2 proteins expression and the *let-7* expression in GEP NETs.

In this study, we investigated the prevalence of HMGA1 and 2 involving in different GEP NETs and assessed its relevance to malignancy. We performed a systematic analysis of immunohistochemistry and reverse transcription (RT)–PCR for HMGA1 and 2 expression in a series of GEP NETs from different sites. *Let-7* was also analysed by semiquantitative RT–PCR. The data were then correlated with relevant clinical information. In addition, we investigated possible associations between HMGA1, 2 expression and *let-7* expression.

## Materials and methods

### Patients and samples

Fifty-five paraffin-embedded surgical or biopsy specimens were collected (from the surgical pathology files of the Department of Pathology, University of Tokushima School of Medicine, and the affiliated hospitals) from patients affected by NETs of GEP from 1982 to 2007. Tumours of the GEP tract were from foregut: esophagous (*n*=1), stomach (*n*=11), pancreas (*n*=11), duodenum (*n*=6); midgut: ileum (*n*=2), appendix (*n*=1); hindgut: colon (*n*=5), rectum (*n*=14); liver metastasis (*n*=1); and lymph node metastasis (*n*=3). The frequency of GEP NETs in this study is general agreement with published reports from the Neuroendocrine Tumour Workshop of Japan (Ito *et al*, 2007). Notably, the frequency of midgut NETs was very low. Paraffin sections were stained with pan-NE markers, such as chromogranin A and synaptophysin, to confirm the NE immunophenotype. The diagnosis of NETs was made on the basis of morphological and immunohistochemical findings evaluated by two independent pathologists (ZRQ and TS), according to the World Health Organization classification (Klöppel *et al*, 2004). These included well-differentiated NET (WNET, of either benign or uncertain behaviour; *n*=29), well-differentiated NEC (WNEC; *n*=13), and poorly differentiated NEC (PNEC; *n*=13). The tumour stage was made on the basis of the recently published TNM classification system (Rindi *et al*, 2006; Rindi *et al*, 2007). Patient characteristics for these cases are summarised in Table 1.

### Immunohistochemistry

High-mobility group A1, HMGA2, and Ki-67 antigen immunolocalisation based on the labelled streptavidin-biotin method were performed on sections from representative blocks of paraffin-embedded tissues. After deparaffinisation and antigen retrieval using an autoclave oven technique, sections were incubated at 4°C overnight with goat polyclonal anti-HMGA1 antibody (1 : 50; Santa Cruz Biotech, Santa Cruz, CA, USA) and goat polyclonal anti-HMGA2 antibody (1 : 50; Santa Cruz Biotech) or with Ki-67 antigen mouse monoclonal antibody (1 : 100, DakoCytomatin, Glostrup, Denmark). Antigen–antibody complexes were detected using the cobalt-3, 3′-diaminobenzidine (Co-DAB) reaction. Squamous cell carcinomas known to be positive for HMGA1 and 2 were used as positive control. Sections incubated in PBS without the primary antibody served as negative controls. Owing to the absence of tissue entirely composed of neuroendocrine cells, several whole normal human organs were used as controls: oesophagus, stomach, duodenum, appendix, small intestine, pancreas, colon, and rectum. Clear nuclear staining was considered to present a positive stain for HMGA1, 2, and Ki-67. A total of 200–1000 cells were counted and the percentage of HMGA1-, 2-stained tumour cells was scored on a scale of 0–3 (0: no expression; 1+: 1–10%; 2+: 10–50%; 3+: >50%). The Ki-67 antigen labelling index (LI) was determined by counting the number of positive cells in a total of 200–1000 tumour cells observed in several representative high-power fields ( × 400).

### Microdissection and RNA isolation

From each paraffin block of representative tumour areas, serial sections with a thickness of 10 *μ*m were prepared and stained with nuclear Fast Red (Sigma-Aldrich, Tokyo, Japan). Malignant cells were selected under microscope magnification ( × 20 to × 100) and dissected from the slide using a scalpel or Laser microdissection (Molecular Machines & Industries, Glattbrugg, Switzerland). Total RNA was isolated using the high pure RNA paraffin kit (Roche Diagnostics GmbH, Mannheim, Germany) as per the manufacturer's instructions. Extracted RNA concentration and purity were measured using a NanoDrop ND-1000 spectrophotometer (NanoDrop Technologies, Del, Wilmington, DE, USA) and by PCR using the primer pair specific for *GAPDH* with or without RT, followed by 1% agarose gel electrophoresis and ethidium bromide staining.

### RT–PCR analysis for HMGA1 and 2

Total RNA (500 ng) was reverse transcribed, using random hexanucleotides as primers (50 pM) and 200 units primescript reverse transcriptase (Takara Bio Inc., Otu, Japan). The cDNA was amplified in a 20 *μ*l reaction mixture containing 0.2 mM dNTP, 1.0 mM MgSO_4_, 0.5 *μ*M each primer and 1 U KOD-Plus polymerase (Toyobo, Osaka, Japan). After a denaturing step (95°C for 2 min), the cDNA was further amplified in 35 PCR cycles (95°C for 1 min, 58°C for 30 s, and 68°C for 30 s). The following primers were used to amplify the HMGA1 transcript: forward primer, 5-AGAGACCTCGGGGCCGACCA-3; reverse primer, 5-GATGCCCTCCTCTTCCTCCTT-3. The following primers were used to amplify the HMGA2 transcript: forward primer, 5-ACTTCAGCCCAGGGACAAC-3, which maps onto the first exon; reverse primer, 5-GCTGCTTTAGAGGGACTCTTGTT-3, which maps onto the second exon. Expression of the *GAPDH* gene was used as an internal control for the amount of cDNA tested. The reaction products were analysed on a 2% agarose gel. Total RNA samples from several whole normal human organs were used as controls: oesophagus, stomach, duodenum, appendix, small intestine, pancreas, colon, and rectum.

### Semiquantitative RT–PCR analysis for *let-7*

The expression of human mature *let-7* (*hsa-let-7*) miRNA was analysed using a *mir*Vana quantitative RT–PCR miRNA detection kit (Ambion, Austin, TX, USA, Cat. nos. AM1558 and AM30000) according to the manufacturer's protocol. Ubiquitously expressed *U6* small nuclear RNA (Ambion, Cat. No. AM30303) was used for normalisation and as an internal control. Briefly, RT–PCR was performed with 20 ng of total RNA using gene-specific RT primers. cDNA was generated. The PCR consisted of 32 cycles (95°C for 15 s, 60°C for 30 s) after an initial denaturation step (95°C for 3 min). The PCR products were analysed by electrophoresis on 2% agarose gels. Quantitation of *let-7* expression levels was achieved by densitometric scanning of ethidium bromide-stained gels. The levels of expression of *let-7* was analysed with computer software (Image, NIH) and shown as the ration of *let-7* to *U6* (Qian *et al*, 2005).

### Statistical analysis

To determine the significance of associations between different variables, data were statistically analysed by Mann–Whitney *U*-test, Kruskal–Wallis test, *χ*^2^–test, and Spearman's correlation coefficient using StatView J-4.5 software (Abacus Concepts, Berkeley, CA, USA). A *P-*value of less than 0.05 was considered statistically significant.

## Results

### HMGA1 and 2 expression analysis by IHC

Immunolocalisation of HMGA1, 2 proteins was mainly nuclear. However, cytoplasmic staining was observed similar to that reported earlier for placental extravillous trophoblast and lung cancer, but was found to be nonspecific background staining and was ignored (Sarhadi *et al*, 2006).

**Figure 1:**
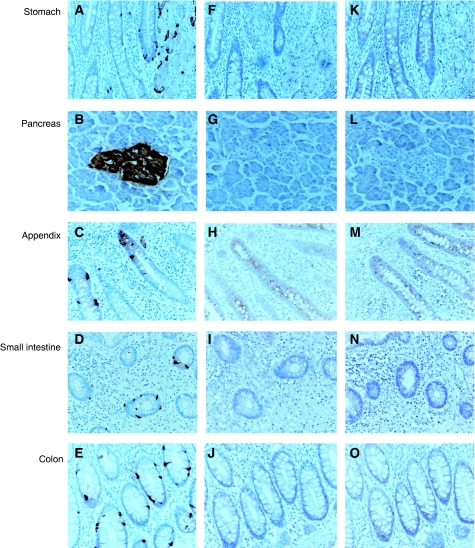
Choromgranin A immunostaining detected normal neuroendocrine (NE) cells in each organ showed cytoplasmic immunoreactions (**A**–**E**). High-mobility group A1 (HMGA1) (**F**–**J**) and HMGA2 (**K**–**O**) showed negative immunoreaction in normal NE cells and other normal cells of each organ.

In general, there were no HMGA1 and 2 expressions in the normal organs including oesophagus, stomach, duodenum, appendix, small intestine, pancreas, colon, and rectum. In GEP normal NE cells, detected by choromgranin A (Figure 1A–E), HMGA1 and 2 were immunostained negatively (Figure 1F–O).

**Figure 2:**
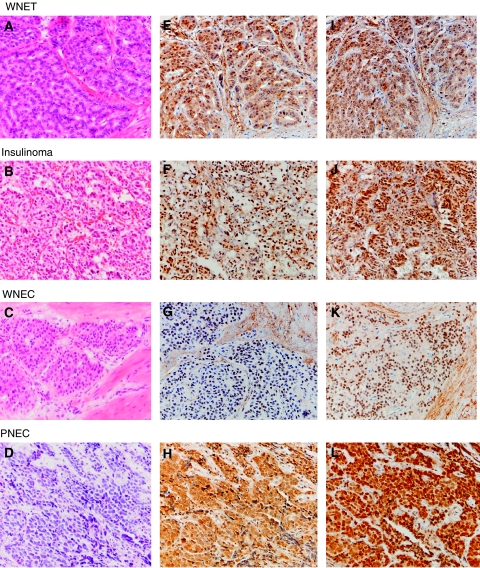
Haematoxilin and eosin staining, high-mobility group A1 (HMGA1) and HMGA2 immunostaining figures of well-differentiated NET (WNET) (**A**, **E** and **I**), insulinoma (**B**, **F**, and **J**), well-differentiated neuroendocrine carcinoma (WNEC) (**C**, **G**, and **K**), and poorly differentiated NEC (PNEC) (**D**, **H**, and **L**), respectively. High-mobility group A1 (**E**–**H**) and HMGA2 (**I**–**L**) showed strongly nuclear immunostaining in gastroenteropancreatic neuroendocrine tumours.

**Figure 3:**
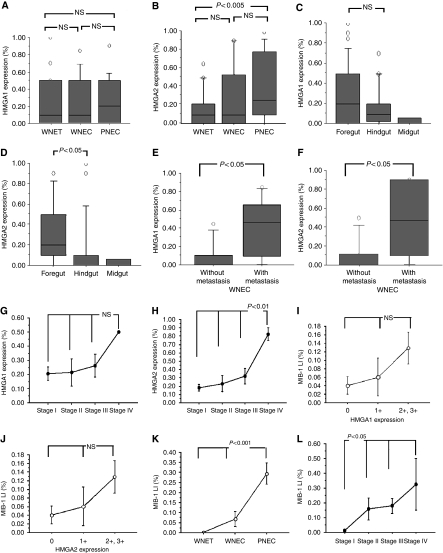
The relations between expression levels of high-mobility group A (HMGA1), HMGA2 proteins and clinicopathologic data of gastroenteropancreatic (GEP) neuroendocrine tumours (NETs). According to different histopathologic categories, HMGA1 protein expression did not show significant difference among well-differentiated NET (WNET), well-differentiated neuroendocrine carcinoma (WNEC), and poorly differentiated NEC (PNEC) (**A**). High-mobility group A2 protein expression increased from WNET to WNEC and PNEC, and its significant difference was observed between WNET and PNEC (*P*<0.005) (**B**). Gastroenteropancreatic NETs in foregut showed the highest level of HMGA1 expression but it was not significant (**C**). Gastroenteropancreatic NETs of foregut also showed the highest level of HMGA2 expression (*P*<0.05) (**D**). In GEP WNECs, the expression of HMGA1 and 2 was higher in metastasis tumours than in tumours without metastasis (*P*<0.05, *P*<0.05) (**E** and **F**). High-mobility group A1 protein expression increased following tumour stage but did not show significant difference (**G**). High-mobility group A2 protein showed the highest level in stage IV GEP NETs (*P*<0.01) (**H**). MIB LI progressively increased following HMGA1 and 2 expression score, although it was not significant (**I** and **J**). MIB-1 LI progressively increased from WNETs to WNECs and PNECs (*P*<0.001) (**K**), and following tumour stage (*P*<0.05) (**L**).

In GEP NETs, a high proportion of tumours (38 of 55, 69%) was detected with nuclear HMGA1 protein expression (Figure 2E–H, Tables 1 and 2). Twenty-seven (49%) tumours showed a high expression (3+ and 2+), 13 (24%) tumours showed a low expression (1+), and 17 (31%) tumours were immuno negative. According to different histopathologic categories, HMGA1 overexpression was detected in 21/29 (72%) of WNETs, 8/13 (61%) of WNECs and 9/13 (70%) of PNECs (Table 2). This HMGA1 protein expression did not show a significant difference level among WNETs, WNECs and PNECs (Figure 3A). However, NETs in foregut showed the highest level of HMGA1 expression even it was not significant (Figure 3C).

A clear nuclear staining for HMGA2 was detected in 40 of 55 (73%) GEP NETs (Figure 2I–L, Tables 1 and 2). Twenty-five of these (46%) showed a high expression (3+ and 2+) of HMGA2, and 15 (27%) tumours showed low expression (1+), and 15 (27%) tumours were negative immunoreactions (Table 2). According to different histopathologic categories, HMGA2 protein expression progressively increased from WNET to WNEC and PNEC, and the significant increasing was detected between WNET and PNEC (*P*<0.005) (Figure 3B). Neuroendocrine tumours of foregut showed the frequent HMGA2 overexpression and highest level of HMGA2 expression (*P*<0.05) (Table 2, Figure 3D).

In WNECs, the expression of HMGA1 and 2 was significantly higher in metastatic tumours than in tumours without metastasis (*P*<0.05, *P*<0.05; respectively; Figure 3E and F). This tendency is not clear in PNECs. In addition, HMGA2 showed the highest level in stage IV GEP NETs (stage IV *vs* stages I, II, III, *P*<0.01; Figure 3H). High-mobility group A1 expression was also higher in stage IV tumours than stages I, II, and III tumours, but it was not significant (Figure 3G).

We also investigated the correlation between MIB-1 labelling index (MIB-1 LI), which was assessed earlier to enroll patients in the clinical trial and HMGA proteins expression. High-mobility group A1 and 2 nuclear staining positively correlated with the proliferation index in GEP NETs (*R*=0.28, *P*<0.05; *R*=0.434, *P*<0.001; respectively). MIB-1 LI progressively increased following HMGA1 and 2 expression score, although not significant (Figure 3I and J). MIB-1 LI also progressively increased from WNETs to WNECs and PNECs (*P*<0.001; Figure 3K) and following tumour stage (*P*<0.05; Figure 3L). Furthermore, MIB-1 LI positively correlated with tumour size (*R*=0.409, *P*<0.005).

Between 6 GI WNETs with uncertain behaviour (>1 cm or vascular invasion) and 14 GI WNETs with benign behaviour, HMGA1 and 2 expressions did not show any significant differences (data not shown). Although HMGA1 and 2 expressions were potentially high in WNECs, there was no significant correlation between HMGA proteins expression and tumour malignancy (i.e., WNETs *vs* WNECs) in foregut or hindgut NETs, respectively (data not shown). We could not find any significant differences in HMGA1 and 2 expression between PNEC areas and adenocarcinoma areas in several PNEC cases (data not shown).

### *HMGA1* and *2* mRNA expression analysis by RT–PCR

**Figure 4:**
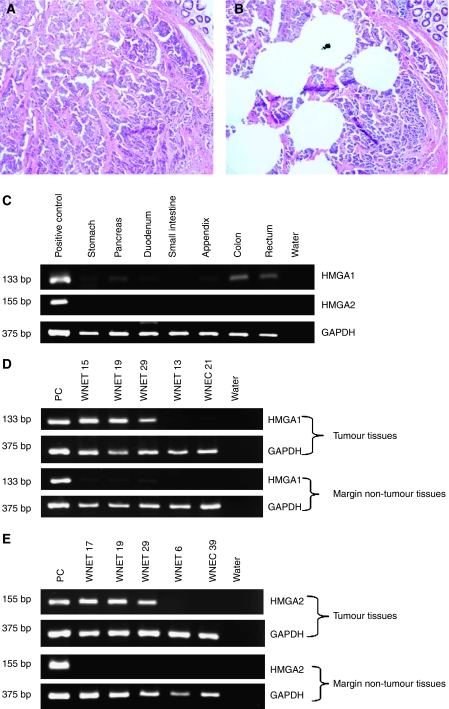
Pre-microdissection figure of one well-differentiated neuroendocrine tumour (WNET) (**A**). Post-microdissection figure of the same WNET (**B**). High-mobility group A1 (HMGA1) (133 bp) and HMGA2 (155 bp) mRNA amplification were not observed in normal tissues. In colon and rectum, a very weak band was detected (**C**). But in tumours, HMGA1 (**D**) and 2 (**E**) mRNA was abundantly amplified, whereas their expressions were not detected in tumour margin non-tumour tissues. *GAPDH* (375 bp) expression was used as endogenous control for RNA integrity.

In RT–PCR mRNA study, *HMGA1* and *2* mRNA expression was analysed on normal tissues from 7 organs (each organ sample pool included RNA from four normal tissues) and 31 tumours with 25 surrounding non-tumour tissues. As shown in Figure 4C, HMGA1 and 2 mRNA amplification was not observed in normal organs. Only a very weak band could be seen in colon and rectum. However, in many tumours HMGA1 and 2 mRNA was abundantly amplified, whereas their expression was not detected in surrounding non-tumour tissues (Table 1, Figure 4D and E). Generally, the mRNA expression of *HMGA1*and *2* correlated well with the nuclear expression of HMGA1 and 2 proteins as detected by IHC analysis (*P*<0.0005, *P*<0.0005, respectively).

### Expression of *let*-7 in GEP NETs

**Figure 5:**
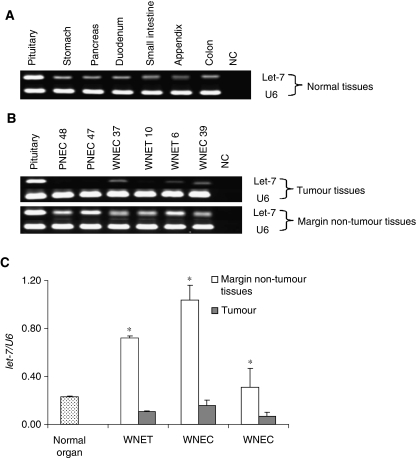
Semiquantitative reverse transcription–PCR method was used to address the levels of *let-7* in normal tissues from 6 organs (stomach, pancreas, duodenum, small intestine, appendix, and colon), 30 gastroenteropancreatic neuroendocrine tumours (NETs), and their margin non-tumour tissues. Normal pituitary tissue was used as positive control. *U6* expression was used as endogenous control for small RNA integrity and as templates for quantitative analysis. Generally, normal tissues from different organs clearly showed *let-7* expression (**A**). Comparing with the level of *let-7* expression in tumour's margin non-tumour tissues, negative or reduced expression of *let-7* was detected in tumours (**B**). The average level of *let-7* expression was significantly lower in well-differentiated NET (WNETs), well-differentiated NECs (WNECs), and poorly differentiated NEC (PNECs) than in their margin non-tumour tissues and in normal organs (^*^P<0.05). Interestingly, the *let-7* expression was higher in tumour's margin non-tumour tissues than in normal organs. In addition, *let-7* expression was not significantly different among WNETs, WNECs, and PNECs (**C**).

Semiquantitative RT–PCR method was used to address the levels of *let-7* in normal tissues from six organs including stomach, pancreas, duodenum, small intestine, appendix, and colon (each organ sample pool included RNA from four normal tissues). Normal pituitary, used as positive control, expressed easily detectable levels of *let-7* (*let-7/U6*, 0.9) (Figure 5A). Other normal samples from six organs showed moderate levels of *let-7* (*let7/U6*, 0.21–0.24) (Figure 5A). This difference may relate to tissue specificity. In tumour margin tissue samples, 22 of 24 cases expressed high levels of *let-7* (*let-7/U6*, 0.33–1.49) (Table 1, Figure 5B). On the basis of the above observation, we arbitrarily classified expression levels of less than one-half of *let-7* value in normal tissues, that is, *let-7*/*U6* <0.1 as significant reduction. Thus, downregulated expression of *let-7* was found in 19 of 30 (63%) tumours (negative, 11; significantly reduction, 9). The average levels of *let-7* expression were significantly lower in GEP NETs than normal organs and their surrounding non-tumour tissues (Figure 5C). *Let-7* expression was not significantly different among WNETs, WNECs, and PNECs (Figure 5C, Table 3).

We also investigated the relationship between *let-7* expression and HMGA1, 2 proteins expression in 30 GEP NETs through several viewpoints. High-mobility group A1 high expression was more frequently detected in 19 tumours with significant *let-7* downregulation (53%) than in tumours with regular level of *let-7* expression (*P*<0.05). The average level of *let-7* expression was lower in NETs with HMGA1 overexpression than in tumours with negative or moderate expression of HMGA1 (data not shown). These data implied that there might be inverse correlation between the expression of *let-7* and HMGA1. Unfortunately, we could not observe the clear inverse correlation between the expression of *let-7* and HMGA2 protein expression, although high expression of HMGA2 was frequently detected in tumours with significant downregulation of *let-7* (42%). When analysed the relation between clinicopathological characteristics of 30 GEP NETs and reduced *let-7* expression, there were no notable differences in patient age, sex, tumour size, vascular invasion, or tumour metastasis (data not shown).

## Discussion

Gastroenteropancreatic NETs originate from the cells of the diffuse endocrine system. The molecular genetic mechanism of development and progression is complex and remains largely unknown. Gastroenteropancreatic NETs do not show alterations in oncogenes, such as *ras, myc, fos jun,* and *src*, or in common tumour suppressor genes (Delle Fave and Corleto, 2001). High-mobility group A1 and 2 have been detected in many kinds of benign and malignant tumours (Fusco and Fedele, 2007). Interestingly, overexpression of HMGA1 and 2 also has been shown in NETs including small cell carcinomas and pituitary adenomas (Brenner *et al*, 2004; Fusco and Fedele, 2007). These findings enhanced us to investigate the role of HMGA1 and 2 protein in GEP NETs tumourigenesis and progression.

Our study is the first time to characterise HMGA1 and 2 overexpression in GEP NETs. We found that HMGA1 and 2 overexpression was a common event in GEP NETs. Notably, a high level overexpression of HMGA1 and 2 was frequently observed in WNETs as in WNECs and PNECs. High-mobility group A proteins contributed to tumourigenesis in various benign human tumours including lipomas, uterine leiomyomas, and pituitary adenomas. Little is known about pathogenesis of sporadic WNETs (Zikusoka *et al*, 2005). Our findings suggested that HMGA proteins might relate to the tumourigenesis of GEP NETs.

High-mobility group A1 and 2 interact with many different transcription factors and influence numerous gene expression patterns. They are important regulators of cell growth, differentiation, apoptosis, and transformation (Reeves, 2001). It has been shown that a high expression of HMGA1 and 2 is associated with a highly malignant phenotype and is a poor prognostic index (Fusco and Fedele, 2007). The expression of HMGA1 differed from the expression of HMGA2 in GEP NETs only with respect to the correlation of expression with histopathologic categories. High-mobility group A2 protein expression increased from WNETs to WNECs and PNECs and from stages I, II, III tumours to stage IV tumours. Interestingly, both HMGA1 and 2 overexpression may relate to tumour metastasis in WNECs. Furthermore, we observed the correlation between cell proliferation marker MIB-1 LI and HMGA protein overexpression. Because more than a half of GEP NETs are functionally inactive and are usually diagnosed once signs and symptoms of tumour metastasis occur (Oberg, 2002), the distinction benign and malignant GEP NET is very important. However, it has not been completely resolved (Rindi and Bordi, 2005). Our data implied that HMGA proteins may reliably predict the course of this disease and may have prognostic significances in GEP NETs. However, the exact mechanism involving in HMGA oncogenic activity in GEP NET needs further investigation.

The current literature suggests that several genes are involved in GEP NET tumourigenesis with significant differences among tumours of different embryological derivatives: foregut, midgut, and hindgut. The MEN1 gene is involved in initiation of 33% of foregut GEP NETs (Lubensky and Zhuang, 2007). 18q defects are present almost exclusively in mid/hindgut NETs (Lubensky and Zhuang, 2007). X-chromosome markers are associated with malignant behaviour in foregut tumours only (Lubensky and Zhuang, 2007). In this study, GEP NETs in foregut showed the highest level of HMGA1 and 2 proteins expression. However, the differences in HMGA protein expression among tumour sites need further investigation in large series of GEP NETs.

Recent studies have shown that *let-7* plays a role as tumour suppressors by negatively regulating expression of *RAS* and *HMGA2* oncogenes (Johnson *et al*, 2005; Hebert *et al*, 2007; Mayr *et al*, 2007; Wang *et al*, 2007). *Let-7* downregulation is commonly seen in neoplasm, including lung, breast and gastric cancers, and uterine leiomyomas (Takamizawa *et al*, 2004; Iorio *et al*, 2005; Wang *et al*, 2007; Motoyama *et al*, 2008). In this study, we observed that *let-7* downregulation is very common from benign NETs to small cell carcinomas. This is the first finding that aberrant expression of *let-7* may relate to GEP NET tumourigenesis. Furthermore, the reverse correlation between *let-7* downregulation and HMGA1 overexpression has been first observed in tumours. Till now, the mechanism of HMGA1 overexpression is not clear and many studies only focus on HMGA2 regulated by *let-7* family (Takamizawa *et al*, 2004; Iorio *et al*, 2005; Wang *et al*, 2007; Motoyama *et al*, 2008). Our findings implied that loss or reduction of *let-7* might be one potential mechanism of HMGA1 protein overexpression. On the other hand, although the clearly reverse correlation between *let-7* downregulation and HMGA2 overexpression was not detected in this small series of GEP NETs, loss and reduction of *let-7* expression may also be one important mechanism of HMGA2 overexpression. The truncated transcripts of HMGA2 with a partial or complete loss of *let-7* complementary sites can explain the increased expression of HMGA2 in tumours with slight reduction or regular level of *let-7* expression (Fusco and Fedele, 2007). In addition, by computer searches, we found that HMGA1 and 2 can be potentially regulated by many other miRNAs. Further investigation of miRNAs would be important in GEP NET tumourigenesis study.

Several studies have aimed to develop cancer therapy by inhibiting HMGA proteins (Scala *et al*, 2000). In pancreatic adenocarcinoma, blocking HMGA protein synthesis has a negative effect on tumour cell proliferation and metastatic potential (Liau *et al*, 2006). The differential expression of HMGA1 and 2 in GEP NETs and normal cells should allow for the specificity and lower toxicity of such therapy. Furthermore, as HMGA1 and 2 proteins are frequently overexpressed in GEP NETs, HMGA-targeted anticancer therapy could have a wide ranged application.

## Figures and Tables

**Table 1:** Summary of characteristics of 55 GEP NETs and expressions of HMGA1, 2, and *let-7*

**Number**	**Sex/ age**	**Location**	**Organ**	**Classification**	**Tumour stage, invasion, or metastasis**	**Tumour size (cm)**	**MIB-1 LI (%)**	**HMGA1 IHC (%)**	***HMGA1* mRNA**	**HMGA2 IHC (%)**	***HMGA2* mRNA**	***let-7* tumour**	***let-7* non- tumour**
1	M/47	Hindgut	Rectum	WNET	Stage Ia	0.4	0	5	ND	10	ND	ND	ND
2	M/40	Foregut	Stomach	WNET	Stage I	0.9	0	0	−	10	+	0	0.71
3	M/75	Foregut	Stomach	WNET	Stage 0	0.3	0	0	ND	18	ND	ND	ND
4	F/70	Foregut	Stomach	WNET	Stage I	0.9	0	0	−	0	−	0	1.2
5	F/62	Hindgut	Rectum	WNET	Stage Ib	1.2	0	5	+	10	+	0.48	0.53
6	F/62	Hindgut	Rectum	WNET	Stage Ib	1.5	1	10	+	0	−	0.1	1.49
7	M/58	Foregut	Stomach	WNET	Stage I	0.8	1	15	+	18	+	0.03	ND
8	M/62	Hindgut	Rectum	WNET	Stage Ia, ly(+)	0.9	0	50	+	25	+	0	0.51
9	M/61	Hindgut	Rectum	WNET	Stage IIa	2.2	0	15	+	0	−	0	1.22
10	F/67	Hindgut	Rectum	WNET	Stage Ia	0.6	0	50	+	0	+	0.06	ND
11	M/56	Hindgut	Rectum	WNET	Stage Ib, ly(+)	1	0	0	ND	0	ND	ND	ND
12	M/34	Hindgut	Rectum	WNET	Stage Ia	0.6	0	70	ND	10	ND	ND	ND
13	M/46	Hindgut	Rectum	WNET	Stage Ia	0.3	0	0	−	0	−	0	0.18
14	F/51	Hindgut	Rectum	WNET	Stage Ia	0.8	1	10	−	10	+	0.3	ND
15	M/62	Hindgut	Rectum	WNET	Stage Ia	0.5	1	20	+	10	+	0	0.47
16	M/70	Foregut	Pancreas	WNET	Stage I	1	0.5	0	+	10	+	0	0.4
17	M/63	Foregut	Pancreas	WNET	Stage I	1.1	0.5	50	+	50	+	0.22	1.4
18	M/63	Foregut	Pancreas	WNET	Stage I	1.5	1	10	+	25	+	0.08	0.85
19	F/48	Foregut	Pancreas	WNET	Stage I	1	0	20	+	50	+	0.3	0.34
20	M/74	Foregut	Pancreas	WNET	Stage I	0.2	1	50	+	0	+	0	1.04
21	M/82	Foregut	Pancreas	WNET	Stage I	1.1	0.5	0	−	25	+	0.09	0.97
22	M/49	Hindgut	Rectum	WNET	Stage Ia	0.4	0.6	5	−	10	+	0	0.49
23	M/35	Hindgut	Rectum	WNET	Stage Ib	1.4	0.4	5	−	10	+	0.12	0.94
24	F/57	Foregut	Pancreas	WNET	Stage I	1.6	2	10	+	20	+	0.33	ND
25	F/38	Hindgut	Colon	WNET	Stage Ia	0.2	0	15	ND	0	ND	ND	ND
26	F/61	Foregut	Duodenum	WNET	Stage I	0.3	0.5	50	ND	65	ND	ND	ND
27	M/64	Foregut	Duodenum	WNET	Stage I	0.5	0	100	ND	20	ND	ND	ND
28	M/53	Foregut	Pancreas	WNET	Stage I	0.6	0.6	50	+	50	+	0.08	0.35
29	F/53	Foregut	Pancreas	WNET	Stage I, v(+)	1.5	0	0	−	10	+	0.1	0.57
30	M/44	Hindgut	Rectum	WNEC	Stage Ia	0.8	0	0	ND	0	ND	ND	ND
31	M/48	Foregut	Metastasis	WNEC	Stage IIIb, ly(+), m(ln)	0.9	3	20	+	10	+	0.001	0.35
32	F/74	Midgut	Appendix	WNEC	Stage IIa	1.3	0	0	ND	20	ND	ND	ND
33	F/69	Foregut	Duodenum	WNEC	Stage IIIb, ly(+), m(ln)	4	0	50	ND	55	ND	ND	ND
34	F/63	Foregut	Pancreas	WNEC	Stage IIa	3	0.5	5	ND	10	ND	ND	ND
35	M/82	Midgut	Ileum−caecum	WNEC	Stage IIa	2.4	0	20	ND	0	ND	ND	ND
36	M/53	Hindgut	Metastasis	WNEC	Stage IIIb, ly(+), m(ln)	2.5	10	50	+	23	+	0.55	1.18
37	F/53	unknow	Metastasis	WNEC	Stage IIIb, ly(+), m(ln)	1.9	10	10	+	85	+	0.19	ND
38	F/68	Foregut	Duodenum	WNEC	Stage IIA	1.5	0	60	+	65	+	0	ND
39	M/66	Foregut	Pancreas	WNEC	Stage IIb	7	5	0	+	0	−	0.03	1.56
40	M/56	Foregut	Duodenum	WNEC	Stage IIIb, ly(+), m(ln)	1.3	50	90	ND	85	ND	ND	ND
41	F/40	Midgut	Metastasis	WNEC	Stage IIIb, m(ln)	1.5	8	0	ND	0	ND	ND	ND
42	M/60	Midgut	Ileum–caecum	WNEC	Stage I	0.6	2	0	ND	0	ND	ND	ND
43	M/50	Foregut	Stomach	PNEC	Stage IIa	0.2	50	90	ND	90	ND	ND	ND
44	F/69	Foregut	Stomach	PNEC	Stage IV, m(li)	0.2	15	50	ND	75	ND	ND	ND
45	F/62	Hindgut	Rectum	PNEC	Stage IA	0.3	30	40	ND	100	ND	ND	ND
46	M/71	Hindgut	Colon	PNEC	Stage IIIb, ly(+), v(+), m(ln)	7	30	0	−	10	+	ND	ND
47	F/53	Hindgut	Sigmoid	PNEC	Stage IIa	2.3	50	20	+	26	+	0	0.07
48	M/72	Foregut	Stomach	PNEC	Stage IIIa, ly(+), v(+)	4.5	0	0	−	65	+	0	0
49	M/69	Foregut	Esophagus	PNEC	Stage IV, m(li)	4.5	50	50	ND	90	ND	ND	ND
50	F/60	Foregut	Stomach	PNEC	Stage IIIb, ly(+), v(+), m(ln)	2.8	20	0	ND	0	ND	ND	ND
51	M/67	Foregut	Stomach	PNEC	Stage IIIb, ly(+), v(+), m(ln)	7	10	20	ND	20	ND	ND	ND
52	M/63	Foregut	Duodenum	PNEC	Stage IIIa, ly(+), v(+)	8	50	25	ND	35	ND	ND	ND
53	M/69	Foregut	Stomach	PNEC	Stage IIIb, ly(+), m(ln)	5.5	25	50	ND	0	ND	ND	ND
54	M/74	Foregut	Stomach	PNEC	Stage IIb, v(+)	5	50	5	ND	10	ND	ND	ND
55	M/57	Hindgut	Colon	PNEC	Stage IIa, ly(+), v(+)	6	3	0	−	10	−	0.19	0.85

GEP=gastroenteropancreatic; NET=neuroendocrine tumour; WNET=well-differentiated neuroendocrine tumour; WNEC=well-differentiated neuroendocrine carcinoma; PNEC=poorly differentiated neuroendocrine carcinoma; v (+)=vascular invasion; ly (+)=lymph vessels invasion; m=metastasis; ln=lymph node; li,=liver; tumour=tumour tissue only; non-tumour=tumour margin non-tumour tissue.

**Table 2:** HMGA1 and 2 proteins expression in GEP NETs and clinicopathologic associations

	**No. of cases**	**HMGA1 expression**			**HMGA2 expression**		
**Variable**	**55**	**Negative (*n*=17)**	**Low (*n*=11)**	**High (*n*=27)**	**Negative (*n*=15)**	**Low (*n*=15)**	**High (*n*=25)**
Age (year)		60.9	56.2	60.3	59.8	54.2	62.4
							
*Gender*							
Female	20	5 (25%)	6 (30%)	9 (45%)	6 (30%)	4 (20%)	10 (50%)
Male	35	12 (34%)	5 (14%)	18 (52%)	9 (26%)	11 (31%)	15 (43%)
							
*Histologic type*							
WNET	29	8 (27.5%)	8 (27.5%)	13 (45%)	8 (27.5%)	10 (34.5%)	11(38%)
WNEC	13	5 (39%)	2(15%)	6 (46%)	5 (39%)	2 (15%)	6 (46%)
PNEC	13	4 (30%)	1 (8%)	8 (62%)	2 (15%)	3 (23%)	8 (62%)
							
*Site*							
Foregut	30	9 (30%)	4 (13%)	17 (57%)	5 (17%)	6 (20%)	19 (63%)^*^
Hindgut	21	5 (24%)	7 (33%)	9 (43%)	7 (33%)	9 (43%)	5 (24%)
Midgut	4	3 (75%)	0	1(25%)	3 (75%)	0	1 (25%)

GEP=gastroenteropancreatic; NET=neuroendocrine tumour; WNET=well-differentiated neuroendocrine tumour; WNEC=well-differentiated neuroendocrine carcinoma; PNEC=poorly differentiated neuroendocrine carcinoma.

Negative=0; Low=1+; High=2+ and 3+; (0: no expression; 1+: 1–10%; 2+: 10–50%; 3+: >50%).

^*^P<0.05.

**Table 3:** *let-7* expression in GEP NETs

		***let-7* expression**		
**Variable**	**Case numbers 30**	**−, ± (*n*=19)**	**+ (*n*=11)**	***P*-value**
*HMGA1*				
Negative	9	7 (36%)	2 (18%)	<0.05
Low	8	2 (11%)	6 (55%)	
High	13	10 (53%)	3 (27%)	
				
*HMGA2*				
Negative	7	6 (31%)	1 (9%)	0.31
Low	10	5 (26%)	5 (45.5%)	
High	13	8 (42%)	5 (45.5%)	
				
*Histologic type*				
WNET	22	14 (64%)	8 (36%)	0.98
WNEC	5	3 (60%)	2 (40%)	
PNEC	3	2 (66.6%)	1 (33.3%)	

GEP=gastroenteropancreatic; NET=neuroendocrine tumour; WNET=well-differentiated neuroendocrine tumour; WNEC=well-differentiated neuroendocrine carcinoma; PNEC=poorly differentiated neuroendocrine carcinoma; −=negative; ±=significant downregulation; +=normal level; Negative=0; Low=1+; High=2+ and 3+.
